# ACNet: An Attention–Convolution Collaborative Semantic Segmentation Network on Sensor-Derived Datasets for Autonomous Driving

**DOI:** 10.3390/s25154776

**Published:** 2025-08-03

**Authors:** Qiliang Zhang, Kaiwen Hua, Zi Zhang, Yiwei Zhao, Pengpeng Chen

**Affiliations:** 1 School of Computer Science and Technology, China University of Mining and Technology, Xuzhou 221116, China; lb19170006@cumt.edu.cn (Q.Z.); zizhang@cumt.edu.cn (Z.Z.); chenp@cumt.edu.cn (P.C.); 2 Mine Digitization Engineering Research Center of the Ministry of Education, Xuzhou 221116, China; 3 Shenzhen Research Institute, China University of Mining and Technology, Shenzhen 518057, China; 4 School of Mechanical, Electronic and Control Engineering, Beijing Jiaotong University, Beijing 100044, China; 22221308@bjtu.edu.cn

**Keywords:** semantic segmentation, deep learning, attention mechanism, convolution, vehicle-mounted cameras, autonomous driving

## Abstract

In intelligent vehicular networks, the accuracy of semantic segmentation in road scenes is crucial for vehicle-mounted artificial intelligence to achieve environmental perception, decision support, and safety control. Although deep learning methods have made significant progress, two main challenges remain: first, the difficulty in balancing global and local features leads to blurred object boundaries and misclassification; second, conventional convolutions have limited ability to perceive irregular objects, causing information loss and affecting segmentation accuracy. To address these issues, this paper proposes a global–local collaborative attention module and a spider web convolution module. The former enhances feature representation through bidirectional feature interaction and dynamic weight allocation, reducing false positives and missed detections. The latter introduces an asymmetric sampling topology and six-directional receptive field paths to effectively improve the recognition of irregular objects. Experiments on the Cityscapes, CamVid, and BDD100K datasets, collected using vehicle-mounted cameras, demonstrate that the proposed method performs excellently across multiple evaluation metrics, including mIoU, mRecall, mPrecision, and mAccuracy. Comparative experiments with classical segmentation networks, attention mechanisms, and convolution modules validate the effectiveness of the proposed approach. The proposed method demonstrates outstanding performance in sensor-based semantic segmentation tasks and is well-suited for environmental perception systems in autonomous driving.

## 1. Introduction

The vigorous development of the intelligent Internet of Things has driven the deep integration of sensors, communication networks, and cloud computing technologies, thereby jointly building a comprehensive and three-dimensional intelligent traffic information interaction network. As a key node, autonomous vehicles utilize multiple intelligent sensors, such as lidar and cameras, to achieve real-time transmission and efficient sharing of traffic data. Within this technical architecture, accurate perception of road conditions and the behavior of other traffic participants has become the cornerstone of safe operation of the autonomous driving system, directly affecting decision-making accuracy and driving safety [1,2,3]. As a fundamental technique in computer vision, semantic segmentation enables accurate localization and identification of multiple objects in road scenes through pixel-level fine-grained classification and annotation. In autonomous driving scenarios, this technology enables rapid differentiation of targets such as vehicles, pedestrians, road boundaries, and traffic lights, while outlining their contours and positions. This allows the autonomous driving system to maintain real-time situational awareness, thereby significantly improving its reliability and safety [4,5,6].

Currently, deep learning-based encoder–decoder architectures have demonstrated excellent performance in the field of semantic segmentation for autonomous driving. The encoder converts the original image pixel information into high-level semantic features and extracts abstract semantic representations. At the same time, the decoder upsamples the low-resolution feature maps and restores spatial details. This process maps semantic features back to their original spatial resolution, enabling accurate pixel-wise prediction. Long et al. [7] proposed the first end-to-end, fully convolutional network for pixel-level prediction, known as FCN. This network discards the dependence of traditional semantic segmentation methods on manual feature extraction, replaces the fully connected layer in the convolutional neural network with a convolutional layer, and solves the problem of fixed image size in traditional neural networks. However, after multiple convolution and pooling processes, the spatial resolution of the feature map continues to decrease, and a large amount of information about small target objects in the image is lost, making it difficult to restore the original contours and details later. To solve the above problems, Ranneberger et al. [8] proposed the classic segmentation network U-Net. This network architecture constructs a symmetric encoder–decoder architecture. The encoder on the left extracts high-level semantic features, while the decoder on the right, aided by skip connections, integrates high- and low-resolution information to enhance segmentation accuracy in target regions. Although this network improves segmentation accuracy, it still has limitations in terms of multi-scale feature extraction and high- and low-level feature fusion complementation. When faced with complex scenes of autonomous driving, the segmentation results are prone to blur and inaccuracy. To overcome this problem, many variant networks have been extended. Zhou et al. [9] introduced U-Net++, which incorporates dense skip connections and nested architectures to reduce the information gap between encoder and decoder, resulting in more effective feature fusion across different levels. Cao et al. [10] proposed the Swin-UNet, which incorporates a transformer-based module into the U-Net to improve the network’s ability to capture global features, demonstrating clear advantages in high-resolution image segmentation.

In autonomous driving applications, to balance the accuracy and computational efficiency of semantic segmentation, the lightweight backbone network MobileNetV2 [11] is often combined with DeepLabv3+ [12]. This architecture achieves real-time and accurate segmentation of targets in road scenes by optimizing feature extraction and multi-scale dilated convolution. However, this method still has certain limitations. On the one hand, although the lightweight design of MobileNetV2 reduces the computational complexity of the model, it may lead to insufficient feature representation capability, especially in complex scenes, making the model more prone to false detections. On the other hand, the dilated convolutions in the DeepLabv3+ may cause grid artifacts with large dilation rates, which weaken spatial detail information. To address the above problems, existing studies have proposed various improvement strategies. Ren et al. [13] replaced the dilated convolution with a depthwise separable convolution. They introduced the shuffle attention mechanism to enhance the model’s ability to capture details and accurately segment object boundaries. In addition, Wang et al. [14] replaced the residual units in the Res2Net backbone with traditional residual blocks to achieve a more detailed multi-scale feature extraction. Furthermore, they designed a multi-level loss function system by constructing loss functions for feature maps at different network depths and their corresponding ground truth labels. These losses were then fused and optimized to enhance the model’s ability to learn multi-scale features.

From the above analysis, we can see that in the application of intelligent Internet of Things, scene understanding ability, feature expression ability, and information interaction ability of deep learning networks are the core elements to ensure the safe operation of intelligent driving systems. Among these, an accurate perception of the global scene enables a vehicle to plan its path, while quick perception of local details is crucial for responding to emergencies. The efficient feature extraction mechanisms for both global and local information form the basis of the model’s ability to learn meaningful information from massive autonomous driving data. However, when dealing with complex environments, existing networks often encounter problems such as an imbalance between global and local information, as well as insufficient feature extraction efficiency. To this end, the method proposed in this paper enhances the understanding of high-dimensional image data captured by vehicle-mounted sensors. By improving feature extraction mechanisms, the model’s perception accuracy and semantic knowledge of its surrounding environment are significantly enhanced, thereby effectively supporting decision-making and control tasks in complex road scenarios for autonomous driving systems. The main contributions of this paper are as follows:

(1) We propose a collaborative network for semantic segmentation tailored to autonomous driving perception tasks. The network effectively integrates two complementary plug-and-play modules to construct a new feature interaction processing mechanism, which improves segmentation accuracy by enhancing cross-scale information collaboration capabilities and multi-branch parallel feature capture capabilities.
(2) We design a novel global–local collaborative attention (GLCA) module, using a dual-path architecture to aggregate global context and local details, as well as incorporating a dynamic attention fusion strategy that adaptively balances global and local features across channels and spatial dimensions, thus achieving an effective trade-off between long-range semantic dependencies and fine-grained target details in complex traffic scenarios.
(3) We propose an innovative spider web convolution (SWConv) module that simulates the spider web topology to obtain six-dimensional context information on the image. This module uses a differentiated padding strategy and multi-dilation rate convolution to integrate multi-view spatial relationships, enhance feature representation capabilities, and improve the perception of irregular target geometric features in complex scenes.
(4) Experimental results on the Cityscapes, CamVid, and BDD100K datasets validate the effectiveness of the proposed GLCA module and SWConv module in the task of semantic segmentation for autonomous driving. Compared with various mainstream attention mechanisms and convolutional structures, the proposed modules achieve state-of-the-art performance across multiple evaluation metrics. Furthermore, when integrated into the baseline model, they demonstrate significant advantages over typical semantic segmentation models.

## 2. Related Work

The network proposed in this paper is based on an encoder–decoder architecture. In semantic segmentation for autonomous driving, recent research has mainly focused on enhancing classical convolutional structures, introducing attention mechanisms, and exploring novel feature interaction strategies. This subsection provides an overview of existing methods based on their design optimization priorities.

### 2.1. Context-Based Network

In semantic segmentation tasks for autonomous driving, context-aware networks enhance segmentation accuracy and overall scene understanding by capturing long-range dependencies between pixels and modeling the scene’s semantic structure. These methods leverage contextual information as a core cue to explore spatial relationships and category dependencies among objects, thereby alleviating issues such as semantic ambiguity and structural discontinuity caused by the limited receptive field of traditional convolution [15,16]. For example, Wu et al. [17] proposed the CGNet semantic segmentation network, which introduces a context guidance module to jointly model local features and contextual information. This effectively enhances the representational capacity of features at each stage, improves the overall segmentation accuracy. Elhassan et al. [18] proposed the DSANet network, which incorporates a spatial encoding network and a dual attention mechanism. It extracts multi-depth features via hierarchical convolution, effectively preserving spatial information while reducing edge detail loss. The dual attention module fuses contextual dependencies in both spatial and channel dimensions to enable more effective integration of semantic and spatial features, thus significantly improving segmentation accuracy. KIM et al. [19] proposed the ESCNet network, which features an efficient spatio-channel dilated convolution module (ESC). By introducing a dilation rate mechanism in the spatial dimension, this module effectively expands the receptive field and enables efficient capture of multi-scale contextual features.

### 2.2. Context Boundary Network

In the field of semantic segmentation for autonomous driving, context-boundary-based networks aim to integrate object edge information with global semantic context. They improve the precision and semantic consistency of segmentation results by constructing cross-level interaction mechanisms between boundary features and scene semantics [20,21]. For example, Xiao et al. [22] proposed the BASeg semantic segmentation network, which includes a boundary refinement module (BRM) and a context aggregation module (CAM). The BRM integrates high-level semantic features with low-level boundary information by combining convolution and attention mechanisms to refine boundary regions. Moreover, it uses semantic features to guide boundary positioning, effectively suppresses noise, and preserves key pixel-level positional information. The CAM focuses on reconstructing long-range dependencies between boundary regions and internal pixels of target objects, thereby enhancing the understanding of feature association. Lyu et al. [23] proposed an edge-guided semantic segmentation network ESNet, which is composed of an edge network, a segmentation backbone network, and a multi-layer fusion module. It first extracts global edge information to enhance its category discrimination capability. Then it integrates the features generated by the segmentation network with edge information at multiple levels in the fusion module, significantly improving segmentation accuracy. Luo et al. [24] proposed a guided downsampling network that uses an autoencoder structure to compress image size while retaining edge, texture, and structural information as much as possible. It adopts a dual-branch encoder architecture to capture long-range dependencies between objects and the global scene layout, providing a strong foundation for pixel-level semantic segmentation in autonomous driving.

### 2.3. Attention and Convolution Optimization

In recent years, attention mechanisms and convolutional optimization techniques have been widely applied to semantic segmentation in autonomous driving. Attention modules enable models to focus on key image regions, thereby enhancing the perception of semantic elements such as roads, vehicles, and buildings. Different types of attention mechanisms vary in scope, implementation, and effectiveness, directly impacting the model’s ability to understand the relationships between multi-scale objects and contextual information in complex road scenes [25,26]. For example, Hu et al. [27] proposed the SE attention mechanism module, which extracts channel features via global information compression and assigns weights to each channel, enhancing the perception of key semantic elements in complex environments. Woo et al. [28] developed the CBAM module, which combines channel and spatial attention using average and max pooling operations to improve feature responses to core objects such as roads and pedestrians while suppressing background noise. Li et al. [29] proposed the SK attention module, which uses parallel multi-scale convolution kernels along with fusion and weighting mechanisms to dynamically select receptive fields, thereby enhancing multi-scale object recognition. Hou et al. [30] introduced the Coordinate Attention module, which incorporates coordinate information through global pooling along horizontal and vertical directions, thereby enabling more precise localization of road targets and improved semantic feature representation. Wang et al. [31] proposed the ECA module, employing one-dimensional convolution to model local channel dependencies while keeping computational costs low. It strengthens core semantic channels such as roads and vehicles, supporting real-time segmentation requirements. Park et al. [32] proposed the BAM module, which jointly models channel semantics and spatial positioning. This facilitates semantic enhancement and precise localization of key targets like road structures and dynamic vehicles, improving the model’s perception in autonomous driving scenarios.

Convolution optimization techniques effectively address challenges such as significant variations in target scale, shape diversity, and complex environments in autonomous driving scenarios by introducing innovative feature extraction methods. For example, Howard et al. [33] proposed depthwise separable convolution, which decomposes standard convolution into depthwise and pointwise operations. This significantly reduces the number of parameters and computational cost, enabling lightweight models to efficiently extract multi-scale features and adapt to the morphological variations of diverse targets, such as vehicles and pedestrians. Chen et al. [34] introduced a dynamic convolution module that generates kernel weights through contextual perception and combines them with spatial convolution to achieve adaptive feature modeling of complex targets. Li et al. [35] proposed the involution module, which uses position-sensitive dynamic kernels to balance fine-grained local modeling and global context extraction. This design enhances the model’s recognition accuracy for complex road conditions and small-scale targets, while also improving its real-time perception capability.

## 3. Materials and Methods

### 3.1. Overall Architecture

This paper adopts the lightweight and efficient MobileNetV2 combined with DeepLabV3+ as the baseline architecture. Owing to its compact size, fast inference speed, and ease of deployment, this architecture is widely used in autonomous driving for scene perception and object segmentation. However, it still faces challenges in complex road scenarios, such as the diverse directional distribution of multi-class targets, blurred boundaries, and the tendency to miss small objects. To address these issues, we introduce the GLCA and SWConv module. These enhancements improve the network’s ability to capture directional spatial patterns and to jointly model global and local features, thereby significantly improving segmentation accuracy and edge detail restoration.

**Figure 1:**
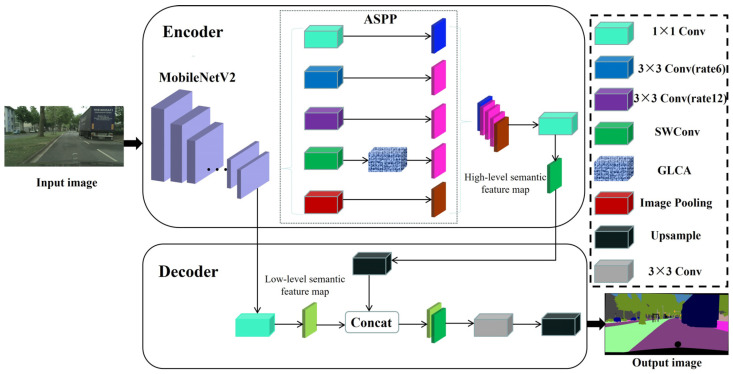
Overall network structure.

As shown in Figure 1, the model adopts an overall encoder–decoder architecture. MobileNetV2 is first used as the backbone network to extract basic semantic features from the input image. Then, the atrous spatial pyramid pooling (ASPP) module from the DeepLabV3+ architecture is introduced to capture contextual information at multiple receptive fields, enhancing the model’s multi-scale feature representation capability. However, the standard ASPP struggles to fully capture multi-directional structural patterns and complex object boundaries in autonomous driving scenarios. To address this, we improve the ASPP module by replacing its final atrous convolution branch with the proposed SWConv module, thereby enhancing directional perception during feature learning. In addition, we incorporate a GLCA module to guide the network in better integrating global semantic information with local details, further improving segmentation accuracy. The enhanced semantic features are then fused with shallow spatial detail features from the encoder to restore spatial resolution. Finally, the decoder progressively upsamples the fused features to produce a pixel-wise segmentation map with the same resolution as the input image, enabling fine-grained perception and segmentation of multi-class objects in autonomous driving scenarios.

### 3.2. Global–Local Collaborative Attention (GLCA) Module

The ASPP module in the baseline model processes feature maps in parallel using dilated convolutions with different sampling rates to capture multi-scale global contextual information, thereby enhancing the model’s semantic understanding of input targets. This module has been widely adopted in various image processing tasks. However, autonomous driving scenarios are significantly more complex than conventional image tasks due to the highly dynamic and diverse road environments. In images captured by onboard cameras, targets vary dramatically in size, shape, color, and position. Under such conditions, the traditional ASPP module demonstrates clear limitations when handling densely distributed and diverse target regions, particularly in capturing fine details of small objects. This can lead to missed or incorrect detections caused by insufficient feature extraction. Moreover, when processing large-scale targets such as vehicles and buildings, ASPP demonstrates limited efficiency in modeling and aggregating global information across diverse scenarios. As a result, it struggles to perceive the complete structure of large objects, leading to poor boundary discrimination and significantly affecting the clarity and accuracy of segmentation outcomes, especially in dynamic environments.

**Figure 2:**
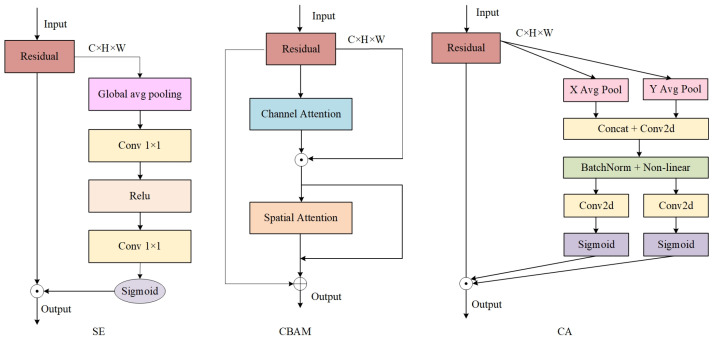
Three types of attention structures.

Currently, various classic attention modules are widely employed to optimize the ASPP component in semantic segmentation tasks for autonomous driving. As illustrated in Figure 2, three commonly used attention mechanisms include the SE module, which focuses on channel information; the CBAM module, which fuses channel and spatial information; and the Coordinate Attention (CA) module. Channel attention mechanisms such as SE, ECA, and SK enhance feature representation by modeling inter-channel dependencies, but they neglect spatial information, resulting in limited perception of critical spatial regions. To address this, modules like CBAM and BAM incorporate spatial attention to improve spatial modeling capabilities. However, they still struggle to balance global semantics and local details in complex scenarios, especially in directional modeling tasks such as lane alignment and vehicle positioning. The CA module introduces a direction-aware mechanism that integrates positional and channel information. Nevertheless, its single-scale structure and static weighting design limit its adaptability to multi-scale targets and dynamic scenes.

**Figure 3:**
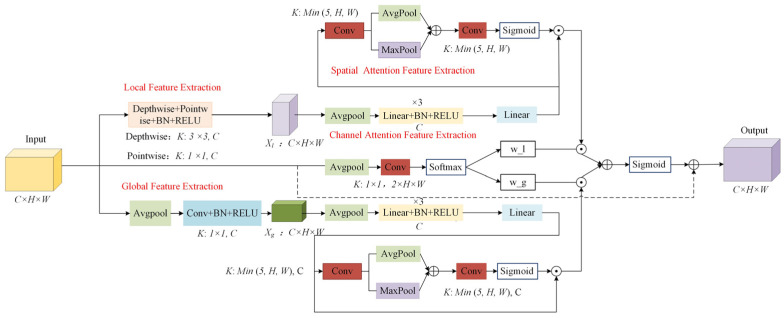
Global–Local Collaborative Attention Module.

To address the limitations of the baseline model and existing attention mechanisms, this paper proposes a novel global–local collaborative attention module, designed to construct an enhanced feature representation framework and comprehensively improve the model’s ability to perceive complex targets. As illustrated in Figure 3, the module adopts a dual-branch architecture consisting of global feature extraction and local detail modeling, specifically tailored to accommodate the significant scale variations commonly observed in autonomous driving scenarios. The input features are first decomposed into global and local components, enabling scale-aware feature learning. In the global branch, adaptive average pooling and convolution operations are applied to reduce spatial resolution and expand the receptive field, thereby enhancing the model’s capability to capture large-scale objects such as buildings and roads. In the local branch, depthwise separable convolutions are employed to extract fine-grained features within each channel without increasing computational complexity. This branch preserves rich spatial and texture details, particularly improving the model’s sensitivity to small objects and their edge structures. Additionally, pointwise convolutions are used to efficiently fuse channel-wise information. Compared to conventional convolutions, depthwise separable convolutions not only reduce parameter overhead but also enhance the extraction of localized features. The detailed implementation of the global and local feature extraction modules is as follows:

(1) $$X_{g}\;=ReLU(BN(Conv_{1\times 1}(AdaptiveAvgPool(X)))),$$

(2) $$X_{l}\;=ReLU(BN(PWConv_{1\times 1}(DWConv_{3\times 3}\;(X)))),$$

where $X\in {\mathbb{R}}^{B\times C\times H\times W}$ is the input feature, $X_{g}$ and $X_{l}$ represent the global feature and local feature after being processed by the feature extraction module.

To further enhance the model’s ability to parse and present features, this paper proposes a channel–spatial attention collaborative learning module that jointly mines the salient information of features. In the designed channel attention module, we employ a three-layer, fully connected network to adequately model the potential nonlinear dependencies among channels. First, the input features are compressed into a global descriptor vector along the channel dimension via global average pooling, followed by a multi-layer, fully connected network to perform nonlinear transformations on this vector. Compared to the traditional SE module, which utilizes a two-layer structure, the three-layer network offers enhanced nonlinear representation capabilities, enabling more effective modeling of complex inter-channel interactions and thereby improving the discriminative power of the attention mechanism in identifying target regions. Furthermore, the three-layer architecture introduces a deeper transformation path during feature compression and recovery, which strengthens the module’s ability to select critical channels and helps suppress background or redundant features that may interfere with the backbone feature flow. Considering the balance between computational efficiency and modeling capacity, we fixed the number of fully connected layers to three. We kept this setting consistent across all experiments without further hyperparameter tuning. However, relying solely on the channel attention mechanism, although effective in enhancing the importance of different channels, inherently overlooks the saliency of features in the spatial dimension. To address this limitation, we further feed the channel-enhanced features into a spatial attention module to achieve a deeper integration of attention mechanisms across both semantic and spatial dimensions. This “channel-first, then spatial” attention strategy enables the model to not only strengthen the representation of key semantic channels but also focus on salient regions within the spatial domain, thereby improving the perception of object locations and boundaries. Specifically, the spatial attention module first applies average pooling and max pooling along the channel dimension of the channel-enhanced feature map to extract spatial response information from different statistical perspectives. These two pooled feature maps are then concatenated along the channel axis and passed through a convolutional layer to generate a spatial attention map. To enhance the module’s adaptability to objects of various scales, the convolution kernel size is dynamically adjusted based on the spatial resolution of the input feature map, allowing the attention mechanism to capture both local details and global structures. Finally, the resulting spatial attention map is activated by a sigmoid function and multiplied with the input feature for spatially selective enhancement. This progressive modeling facilitates complementary fusion of semantic and spatial information, enabling the model to achieve greater robustness and discriminative power in multi-scale recognition. The implementation process is as follows:

(3) $$X_{g\_att\_c}\;=Linear(RELU(BN(Linear(AvgPool(X_{g}))))),$$

(4) $$X_{l\_att\_c}\;=Linear(RELU(BN(Linear(AvgPool(X_{l}))))),$$

(5) $$X_{g\_att}\;=Sigmoid(Conv(Cat(AvgPool(X_{g\_att\_c}),AvgPool(X_{g\_att\_c}))))\times X_{g\_att\_c},$$

(6) $$X_{l\_att}\;=Sigmoid(Conv(Cat(AvgPool({Xl}_{\_att\_c}),AvgPool({Xl}_{\_att\_c}))))\times X_{l\_att\_cl},$$

where features $X_{g\_att\_c}\;$ and $X_{l\_att\_c}$ denote the channel features extracted from the global and local feature branches, respectively. Features $X_{g}$ and $X_{g}$, obtained from Equations (1) and (2), are then fed into the channel attention module to learn channel-wise attention. The resulting channel features are subsequently input into the spatial attention module to capture spatial saliency information, ultimately yielding the final output features $X_{g\_att}\;\;$ and $X_{l\_att}$ from the global and local branches.

Given the varying feature extraction requirements of targets at different scales in autonomous driving scenarios, this paper introduces a dynamic weight allocation module within the attention mechanism, replacing the traditional fixed-weight strategy. The module first applies global average pooling to the input features to extract global semantic information. Then it compresses the feature channels using a 1 × 1 convolution to produce two independent feature weighting branches. Finally, the two branches are normalized using the Softmax function to dynamically generate weighting coefficients for the global and local branches. The specific implementation process is as follows:

(7) $$W=Conv_{1\times 1}(AvgPool(X)),$$

(8) $$W_{global}\;,W_{local}\;=Softmax(W,dim=1),$$

where W, $W_{global}$, and $W_{local}$ represent the original feature weight, global feature weight, and local feature weight, respectively.

After the channel and spatial attention processing, the global–local collaborative attention module performs a weighted fusion of global and local features to generate the final attention weight map. Subsequently, a residual connection is incorporated to adaptively enhance the features, effectively retain key information, and dynamically focus on targets of different scales. The implementation process is as follows:

(9) $$X_{out}=(1+Sigmoid(W_{global}\;⊙X_{g\_att}\;+W_{local\;}⊙X_{l\_att}\;))\cdot X.$$

where feature $X_{out}$ denotes the final feature obtained after learning through the global–local collaborative attention module. Weight tensors $W_{global}$ and $W_{local}$, each with shape $\;\left(B,\;1,\;1,\;1\right)$, represent global-level weighting coefficients that are applied to the corresponding features, $X_{g\_att}\;\;$ and $X_{l\_att}$, through broadcasting. Both $X_{g\_att}\;\;$ and $X_{l\_att}$ features have dimensions $\;\left(B,\;C,\;H,\;W\right)$, indicating batch size, channel number, height, and width, respectively. The operator ⊙ denotes element-wise multiplication, meaning corresponding elements are multiplied one by one to form a new tensor. According to broadcasting rules, tensors of shape $\;\left(B,\;1,\;1,\;1\right)$ are automatically expanded to $\;\left(B,\;C,\;H,\;W\right)$ to enable element-wise multiplication with the feature tensors. After the multiplication and addition operations, a Sigmoid activation function is applied, resulting in a weight tensor of shape $\;\left(B,\;C,\;H,\;W\right)$. This weight is then multiplied element-wise with the original input tensor X to perform weighted fusion, yielding the final output $X_{out}$.

### 3.3. Spider Web Convolution (SWConv) Module

In the context of semantic segmentation for autonomous driving, the surrounding environment is typically highly structured. Yet, it encompasses a diverse range of target categories, including pedestrians, vehicles, lane lines, and traffic signs. These targets exhibit complex directional distributions, drastic scale variations, and blurred or hard-to-distinguish boundaries. Although the ASPP module captures multi-scale contextual information through dilated convolutions, it still faces certain limitations in autonomous driving scenarios. First, when extracting features from target regions, dilated convolutions exhibit uniform perception in all directions and lack apparent directional selectivity. This may lead to inaccurate feature representations when dealing with complex and directionally structured driving scenes, resulting in unclear object boundaries or category confusion. Therefore, a semantic segmentation model for autonomous driving should not only have a sufficiently large receptive field to capture rich contextual information but also possess the ability to sensitively perceive directional structures and accurately delineate edge contours, thereby improving segmentation accuracy and robustness.

To address the above limitations, various convolutional module optimization schemes have been proposed, such as depthwise separable convolution, dynamic convolution, and involution. These methods optimize traditional convolution from different perspectives, such as reducing computational complexity by decomposing standard convolution, introducing dynamic mechanisms for adaptive weight adjustment, and designing spatially specific kernels to enhance the modeling of long-range context. Although these optimizations have improved the expressiveness and efficiency of the model to some extent, they still suffer from insufficient adaptability in the specific context of autonomous driving. Current methods have not fully accounted for the hierarchical differences in multi-scale targets prevalent in autonomous driving scenes, nor the correlations among long-range semantic features. The lack of multi-faceted collaborative feature modeling and contextual semantic perception capabilities makes it difficult to achieve fine-grained feature extraction and learning in complex scenarios.

**Figure 4:**
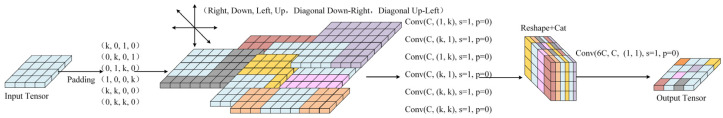
Spider web convolution module. The convolution kernel size (k) is set to 3, and after concatenating the six-directional perception paths, the number of channels becomes 6C. And final 1 × 1 convolution is then applied to reduce the channels back to the original dimension C.

Based on the above problems, this paper proposes a spider web convolution module suitable for complex autonomous driving scenarios with multi-directional perception capabilities, which effectively integrates contextual information from horizontal, vertical, and diagonal directions to enhance feature representation capabilities. In order to retain the multi-scale feature extraction advantages of the ASPP module, this paper finally replaces its last layer of hole convolution with spider web convolution. The spider web convolution is based on directional asymmetry as its core design concept and is inspired by the radial structure of a spider web. It aims to enhance the feature capture capability of convolution in multiple directions, thereby improving the model’s perception of target boundaries and spatial structures. As shown in Figure 4, the module first constructs six asymmetric feature filling strategies to perform multi-directional filling processing on the original input features, namely, top, bottom, left, right, upper left, and lower right. By applying asymmetric padding to the feature boundaries, the receptive field around the edges is effectively expanded, thereby helping to alleviate the problem of edge information loss. In addition, the directional padding design guides the convolutional kernels to focus and perceive in specific directions, thereby enhancing the model’s ability to capture the directional distribution of target structures as well as improving boundary modeling and feature integrity in complex scenes. The implementation process is as follows:

(10) $$P_{right}\;(X)=ZeroPad2d(X;padding=(k,0,1,0)),$$

(11) $$P_{down}\;(X)=ZeroPad2d(X;padding=(0,k,0,1)),$$

(12) $$P_{left}\;(X)=ZeroPad2d(X;padding=(0,1,k,0)),$$

(13) $$P_{up}\;(X)=ZeroPad2d(X;padding=(1,0,0,k)),$$

(14) $$P_{down-right}\;(X)=ZeroPad2d(X;padding=(k,k,0,0)),$$

(15) $$P_{up-left}\;(X)=ZeroPad2d(X;padding=(0,k,k,0)),$$

where $X\in {\mathbb{R}}^{B\times C_{in}\;\times H\times W}$ is the original input feature map, $P_{right}\;(X)$, $P_{down}\;(X)$, $P_{left}\;(X)$, $P_{up}\;(X)$, $P_{down-right}\;(X)$, and $P_{up-left}\;(X)$ represent the original features after padding in different directions, guiding the convolutional kernels to focus and perceive toward the right, bottom, left, top, bottom-right, and top-left, respectively. ZeroPad2d represents zero-fill operation.

After implementing the above-mentioned asymmetric padding processing, the module performs convolution operations on the features after padding in six directions in turn. For the features after padding on the left and right sides, convolution is performed in the horizontal direction to capture the horizontal structural information; for the features after padding on the top and bottom sides, convolution is performed in the vertical direction to extract the longitudinal features; for the features after padding in the upper left and lower right directions, they are processed by standard convolution kernels, so that the convolution kernel can effectively cover and learn the contextual structure in the diagonal direction, thereby enhancing the model’s perception and expression capabilities of diagonal features. In this design, the convolution operation in each direction focuses on the structural pattern in the corresponding direction. Through the separated direction perception strategy, the convolution module can independently learn and strengthen the spatial structural features from six different directions, thereby improving the integrity and directional sensitivity of the overall feature representation.

(16) $$Y_{right}=SiLU(BatchNorm(Conv2d_{1\times k}\;(P_{right}\;(X)))),$$

(17) $$Y_{down}=SiLU(BatchNorm(Conv2d_{k\times 1}\;(P_{down}\;(X)))),$$

(18) $$Y_{left}=SiLU(BatchNorm(Conv2d_{1\times k}\;(P_{left}\;(X)))),$$

(19) $$Y_{top}=SiLU(BatchNorm(Conv2d_{k\times 1}\;(P_{top}\;(X)))),$$

(20) $$Y_{down-right}=SiLU(BatchNorm(Conv2d_{k\times k}\;(P_{down-righ}\;(X)))),$$

(21) $$Y_{up-left}=SiLU(BatchNorm(Conv2d_{k\times k}\;(P_{up-left}\;(X)))),$$

where SiLU is the activation function, BatchNorm is the batch normalization operation, $Y_{right}$, $Y_{down}$, $Y_{left}$, $Y_{top}$, $Y_{down-right}$, and $Y_{up-left}$ are the convolution operations in specific directions applied to the padded features, and the feature maps in six directions obtained by normalization and activation functions.

After completing the convolution operations in the above directions, the feature maps in all directions are restored to the same spatial dimensions as the original input features through bilinear interpolation. Subsequently, a 1 × 1 convolution is used to fuse and splice the features in each direction in the channel dimension, thereby realizing the integration and feature enhancement of multi-directional perception information. This process not only improves the directional selectivity but also effectively enhances the model’s ability to express complex structural information. The specific implementation process is as follows:

(22) $$Y=Concat(Y_{right}^{’},Y_{down}^{’},Y_{left}^{’},Y_{top}^{’},Y_{down-right}^{’},Y_{up-left}^{’}).$$

where $Y_{right}^{’}$, $Y_{down}^{’}$, $Y_{left}^{’}$, $Y_{top}^{’}$, $Y_{down-right}^{’}$, and $Y_{up-left}^{’}$ are feature maps that have been processed to match the size of the output feature map, and Y represents the final output feature.

## 4. Results

### 4.1. Experiment Setup

Datasets: To validate the effectiveness of the proposed modules, we selected three widely recognized and authoritative benchmark datasets in the autonomous driving domain—Cityscapes, CamVid, and BDD100K. Vehicle-mounted sensors collect all three datasets and authentically reflect the visual information captured by camera sensors in urban driving environments, representing diverse types of autonomous driving scenarios and varying visual complexities. By conducting systematic training and evaluation on these absolute sensor datasets, we comprehensively verify the performance and robustness of the proposed method in practical application scenarios, further enhancing its practical relevance and applicability to sensor-based autonomous driving systems.

Cityscapes [36]: The dataset captures urban street scenes from multiple viewpoints, encompassing a variety of architectural styles, road layouts, and traffic conditions. It includes 5000 finely annotated and 20,000 coarsely annotated images. In this study, only the finely annotated images are used, with 2975 images for training. As the official test set does not provide publicly available labels, 500 images from the validation set are used for evaluation, following the common practice in previous works. The resolution of each image in this dataset is 2048 × 1024, and it contains 19 evaluation categories, such as roads, pedestrians, cars, traffic lights, traffic signs, and buildings. To verify the robustness of the model under varying practical scenarios, the input resolution is adjusted. After applying random scaling, cropping, and padding as data augmentation, the final training image sizes are set to 256 × 256 and 768 × 768 pixels, respectively.

CamVid [37]: The dataset is divided into training, validation, and test sets, containing 367, 101, and 233 images, respectively. The original resolution of each image is 480 × 360 pixels. To evaluate the effectiveness of the proposed model and module, the resolution is increased to 960 × 720 pixels through padding. Additionally, data augmentation techniques such as random scaling, cropping, and padding are applied during training to enhance the diversity of the dataset. The dataset includes 11 evaluation categories, covering scene elements such as roads, buildings, trees, and vehicles.

BDD100K [38]: The dataset is a large-scale, multi-task dataset specifically designed to support autonomous driving research. It encompasses a range of computer vision tasks, including image classification, object detection, instance segmentation, panoptic segmentation, and semantic segmentation. This dataset comprises 10,000 images that are fully annotated with pixel-level semantic segmentation labels, encompassing a diverse range of real-world driving scenarios. It captures a diverse set of weather conditions, lighting variations, and road environments, offering extensive scene diversity and reflecting the complexities encountered in actual autonomous driving tasks. The dataset is structured into three distinct subsets, with 7000 images allocated for training, 1000 images designated for validation, and 2000 images reserved for testing. Since the true labels for the test set are not publicly released, the performance evaluation in this study is conducted on the validation set. Initially, the image resolution is 1280 × 720 pixels, which is standard for high-definition video content. However, for this study, the images have been resized to 512 × 512 pixels to fit the model’s training requirements. The dataset contains a total of 19 distinct evaluation categories, each corresponding to specific elements or regions of interest in the driving environment.

Evaluation Metrics: In this study, we evaluate model performance using mean Intersection over Union (mIoU), mean recall (mRecall), mean precision (mPrecision), mean pixel accuracy (mPA), frames per second (FPS), the number of parameters (Params), and floating-point operations (FLOPs). mIoU provides a comprehensive evaluation of the model’s ability to predict semantic categories and their boundaries. mRecall measures the model’s capacity to identify true targets across categories, while mPrecision focuses on the accuracy of predictions, effectively evaluating the model’s ability to reduce false positives. mPA assesses the classification accuracy at the pixel level. FPS (frames per second) represents the number of image frames processed per second and reflects the model’s real-time inference capability. The number of parameters (Params) refers to the total count of learnable weights in the model, reflecting the model’s memory footprint and architectural complexity. FLOPs (floating-point operations) measure the total number of operations required during a single forward pass, providing an estimate of the computational cost and inference efficiency. Specifically, the model’s parameter count is obtained by summing all learnable parameters across layers, while FLOPs are calculated by accumulating the computational cost of each layer based on the model architecture and the dimensions of the input feature maps. The definitions of these metrics are provided as follows:

(23) $$mIoU=\frac{1}{C}\sum_{i=0}^{C-1}\frac{n_{ii}}{\sum_{j=0}^{C-1}n_{ij}+\sum_{j=0}^{C-1}n_{ji}-n_{ii}},$$

(24) $$mRecall=\frac{1}{C}\sum_{i=0}^{C-1}\frac{{TP}_{i}}{{TP}_{i}+{FN}_{i}},$$

(25) $$mPrecision=\frac{1}{C}\sum_{i=0}^{C-1}\frac{{TP}_{i}}{{TP}_{i}+{FP}_{i}},$$

(26) $$mPA=\frac{1}{C}\sum_{i=0}^{C-1}\frac{n_{ii}}{n_{i}},$$

(27) $$FPS=\frac{F}{T}.$$

where *C* is the total number of categories in the image, $n_{ii}$ is the number of pixels correctly classified in the i-th category, $n_{i}$ is the total number of pixels in the i-th category, $n_{ij}$ is the number of pixels that are actually in the i-th category but predicted to be in the j-the category, $TP_{i}$ is the number of pixels in the i-th category correctly predicted as positive samples by the model, $FN_{i}$ is the number of samples in the i-th category that are actually positive but incorrectly predicted as negative samples by the model, $FP_{i}$ is the number of false positives in the i-th category, F is the number of image frames processed in a period of time, and T is the total processing time.

Implementation details: All experiments are conducted on a system equipped with an RTX 4090 GPU using the PyTorch 2.5.0 framework. The initial learning rate is set to 0.0001, and the Adam optimizer is used. Additionally, a cosine annealing decay strategy is introduced to dynamically adjust the learning rate and avoid convergence to local minima. For the loss function, a combination of cross-entropy loss and Dice loss is adopted. During training and testing on the Cityscapes dataset, when the image resolution is adjusted to 768 × 768 pixels, the batch size is set to 4; when the resolution is 256 × 256, the batch size is set to 8. The model is trained for 200 epochs. In the CamVid dataset experiments, when the image resolution is 960 × 720, the batch size is set to 4; when the resolution is adjusted to 480 × 360, the batch size is set to 8. The model is trained for 150 epochs. When conducting experiments on the BDD100K dataset, a batch size of 8 was used, and the model was trained for 200 epochs.

### 4.2. Comparative Experiment

#### 4.2.1. Attention Comparison Experiment

To evaluate the effectiveness of the global–local collaborative attention module proposed in this paper within the autonomous driving scenario, representative classical attention mechanisms currently prevalent in this field were selected for comparison and systematic experimentation. In addition to traditional attention mechanisms, Transformer-based methods, including Swin Transformer and Vision Transformers with Hierarchical Attention, were also considered. Multiple performance metrics, including mIoU, mean recall, mean precision, and mean pixel accuracy, were used to compare and analyze the performance differences of each attention module on the Cityscapes, CamVid, and BDD100K datasets, thereby thoroughly validating the advantages and innovations of the proposed module. Table 1 presents a quantitative comparison of various attention mechanisms on the Cityscapes dataset, which has a resolution of 256 × 256 pixels. The evaluated modules include SE, CBAM, SK, CA, ECA, BAM, Swin Transformer (Swin-TF) [39], and Vision Transformers with Hierarchical Attention (HAT-Net) [40]. All of these modules enhance the model’s ability to focus on key information through design strategies targeting different dimensions such as channels, spatial attention, or scale. Meanwhile, Transformer-based methods further improve the model’s capability to capture long-range dependencies in complex scenarios by modeling global information and leveraging multi-level self-attention mechanisms. These approaches enable the model to focus on critical features over a larger context, distinguishing them from traditional attention mechanisms. To highlight top results, all the best metrics are marked in bold.

To ensure the fairness of the experiment, all attention modules were inserted at the same position within the baseline segmentation model, with consistent experimental environments and hyperparameter settings. As can be seen from the results in Table 1, after integrating different attention mechanisms (including Transformer-based modules) into the ASPP module, the model demonstrated improvements across all four evaluation metrics. Among these, the proposed GLCA module achieved the best performance on all metrics and the most significant improvements. Specifically, the GLCA module improved mIoU by 2.22%, mRecall by 2.11%, mPrecision by 1.58%, and mean pixel accuracy by 0.23%. These quantitative results indicate that the GLCA module significantly enhances semantic segmentation accuracy and classification stability in complex scenes by strengthening multi-dimensional feature interactions and dynamic weighting.

**Figure 5:**
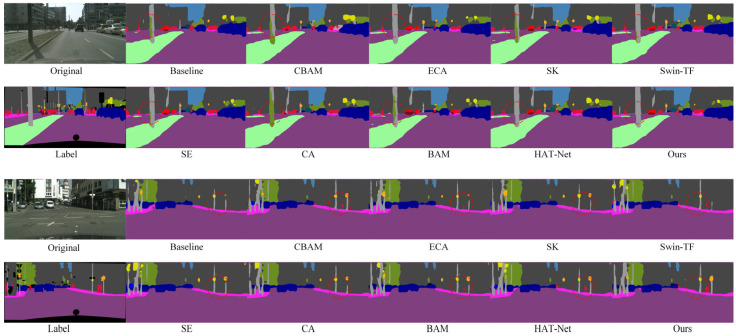
Qualitative comparison of experimental results for different attention and transformer modules on the Cityscapes dataset.

Figure 5 shows a visual comparison of semantic segmentation results after introducing different attention mechanisms and transformer modules. The proposed GLCA module yields superior segmentation in complex scenes compared to the baseline and other attention methods. Especially in the red-circled area, the contours of small targets, such as pedestrians and traffic signs, are more precise and complete, and the misclassification of large targets is significantly reduced, further demonstrating the GLCA module’s advantages in improving segmentation accuracy and robustness.

**Figure 6:**
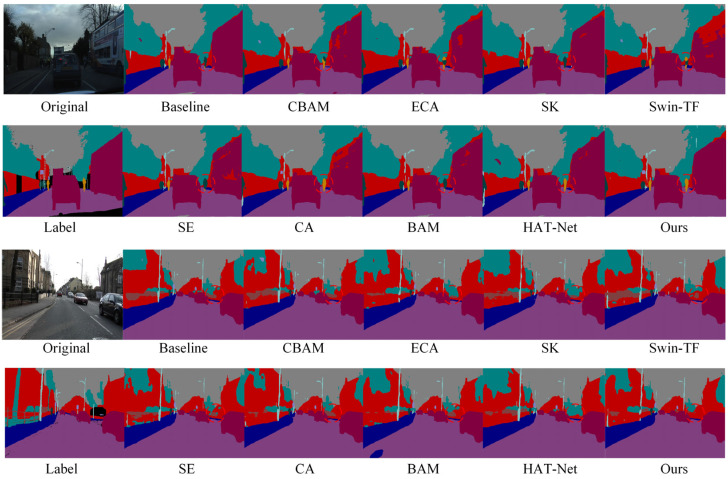
Qualitative comparison of experimental results for different attention and transformer modules on the CamVid dataset.

To further verify the effectiveness of the proposed module in the semantic segmentation task of autonomous driving, we conducted both quantitative and qualitative comparative experiments on the CamVid dataset, which has a resolution of 480 × 360. Table 2 presents the quantitative results obtained by integrating various attention mechanisms into the baseline model. The results show that the GLCA module achieves significant performance improvements on this dataset and outperforms all other attention mechanisms. Figure 6 displays the corresponding qualitative segmentation results. As shown in the red-marked areas, the GLCA module exhibits fewer misclassifications for both small and large objects, and its segmentation outputs are consistent with the ground truth, further demonstrating the module’s robustness and generalization capability in segmentation accuracy.

In real-world autonomous driving scenarios, models are expected not only to perform well on standard datasets but also to demonstrate robustness under complex environmental conditions. To this end, we conducted additional experiments on the BDD100K dataset, which includes diverse weather, lighting, and traffic conditions, to further evaluate the generalization ability of the proposed method in challenging scenes. As shown in Table 3, we present a quantitative comparison of different attention mechanisms and Transformer-based modules on the BDD100K dataset. The results clearly indicate that the proposed GLCA module achieves notable advantages across several key performance metrics. Specifically, compared to the widely used CBAM module, GLCA improves mIoU, mRecall, mPrecision, and mAccuracy by 0.95%, 2.13%, 0.03%, and 0.07%, respectively. Given that BDD100K is a highly challenging dataset characterized by complex and diverse real-world conditions, these improvements underscore the strong robustness and generalization capability of GLCA. Even under such demanding settings, GLCA consistently delivers stable and superior performance, further validating its practical applicability and effectiveness in real-world scenarios.

**Figure 7:**
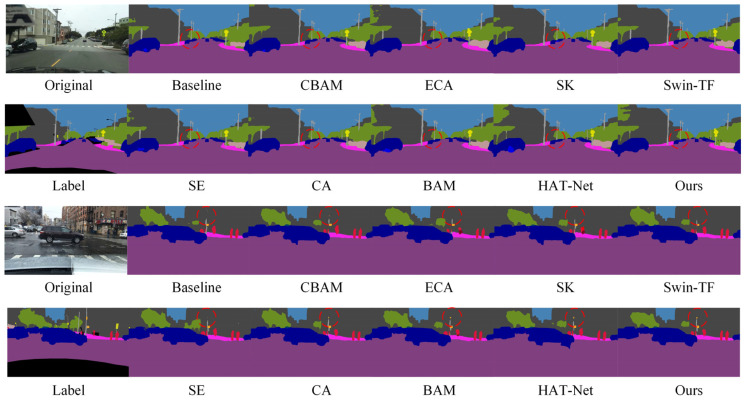
Qualitative comparison of experimental results for different attention and transformer modules on the BDD100K dataset.

In addition to quantitative evaluations, we also conducted qualitative visual analyses on the BDD100K dataset to further assess the performance of each module under complex scenarios. As shown in Figure 7, we compare the segmentation results of different attention mechanisms and Transformer-based modules across various challenging environments. The proposed GLCA module achieves more accurate feature extraction and boundary recognition in critical regions, enabling more precise delineation of essential targets such as road edges, pedestrian contours, and traffic signs. In contrast, other modules often suffer from blurred boundaries or missed detections in certain areas, whereas GLCA consistently maintains strong regional consistency and structural integrity. These results further demonstrate the superior adaptability and robustness of GLCA in handling the complexities of real-world autonomous driving environments, highlighting its potential and effectiveness in practical applications.

To further assess the deployment potential of the proposed GLCA module in practical applications, this paper analyzes the computational costs of models embedding different attention mechanisms and Transformer modules. To further evaluate the deployment potential of the proposed GLCA module in practical applications, this paper analyzes the computational costs of models incorporating various attention mechanisms and Transformer modules. The results are presented in Table 4. Compared to segmentation models that embed different attention mechanisms, the proposed GLCA module introduces minimal computational overhead. Compared with SKAttention, GLCA demonstrates lower computational cost. Moreover, in terms of both parameter count and FLOPs, the GLCA module significantly outperforms models integrating Transformer modules.

Additionally, the GLCA module achieves notable improvements in segmentation performance while maintaining low computational cost, surpassing models with other embedded modules. A comprehensive analysis of Parameters and FLOPs indicates that the GLCA module effectively controls model complexity while maintaining high segmentation accuracy, demonstrating superior computational resource efficiency and design advantages. This module significantly reduces the computational burden of the model, thereby lowering resource consumption during inference and facilitating the demands for lightweight and efficient deployment in practical applications. Therefore, the GLCA module exhibits strong adaptability and potential for broad adoption, making it especially suitable for embedded systems and edge computing environments where computational resources are limited and real-time performance is crucial.

#### 4.2.2. Convolution Comparison Experiment

To verify the adaptability of the spider web convolution proposed in this paper in complex autonomous driving scenarios, we selected several representative convolution methods for comparative experiments, including depthwise separable convolution (DSConv), dynamic convolution (DYConv), inner convolution (INNConv), TVConv [41], and Snake Convolution (DSCConv) [42]. The specific method is to replace the last layer of the hole convolution in the ASPP module with the above different types of convolution structures. All experiments are conducted under the same operating environment and hyperparameter settings to ensure fairness.

The quantitative experimental results on the Cityscapes dataset are shown in Table 5. The results demonstrate that the proposed convolution modules improve the performance of the baseline model to varying extents. Among them, the spider web convolution proposed in this paper achieves the best performance across multiple key metrics. After introducing the spider web convolution, the mIoU increased by 2.35%, mRecall by 2.62%, mPrecision by 1.45%, and mAccuracy by 0.27%, compared to the baseline model. These results indicate that the spider web convolution, through its multi-directional feature perception and fusion design, significantly enhances the model’s ability to extract structural features, thereby improving overall semantic segmentation performance. Compared with other convolution methods, the spider web convolution demonstrates stronger adaptability in accurately recognizing diverse targets such as road boundaries, traffic signs, vehicles, and pedestrians, highlighting its practical value in complex autonomous driving scenarios.

**Figure 8:**
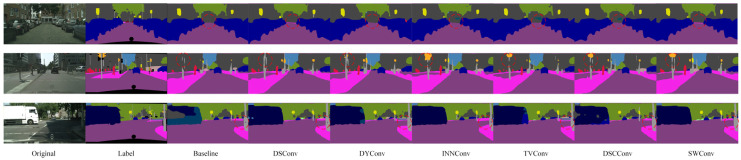
Qualitative comparison experimental results of different convolution modules on the Cityscapes dataset.

Figure 8 shows a visual comparison of the segmentation results of the model after replacing different convolution modules on the Cityscapes dataset. It can be clearly seen that compared with the baseline model, after the introduction of various convolution modules, the segmentation effect of the model in multiple key areas has been significantly improved: the continuity of road boundaries is stronger, the outlines of traffic signs are clearer, the edge details of targets such as vehicles and pedestrians are more accurate, and the perception of overall multi-directional structural features is significantly enhanced. In particular, in the position marked by the red circle in the figure, it can be seen that the model using spider web convolution is more accurate in the recognition and segmentation of targets such as roads, traffic signs, and pedestrians, and the generated results are more delicate and highly consistent with the real labels, which further verifies the effectiveness and advantages of spider web convolution in processing complex structural features.

To verify the effectiveness and generalization ability of the proposed module across different scenarios, we conducted a similar comparative experiment on the CamVid dataset. Table 6 presents the quantitative evaluation results after replacing the dilated convolution in the ASPP module with different convolution methods. The results show that substituting the original dilated convolution with the spider web convolution leads to improvements in all metrics: mIoU increases by 1.02%, mRecall by 0.35%, mPrecision by 1.20%, and mAccuracy by 0.26%. Notably, when other classic convolution modules are used as replacements, the model’s performance on this dataset declines, suggesting that these methods are less effective in capturing fine-grained features and adapting to complex environments. These findings further demonstrate the advantages of spider web convolution in complex semantic segmentation tasks, highlighting its superior feature modeling capability and robustness across diverse scenarios.

**Figure 9:**
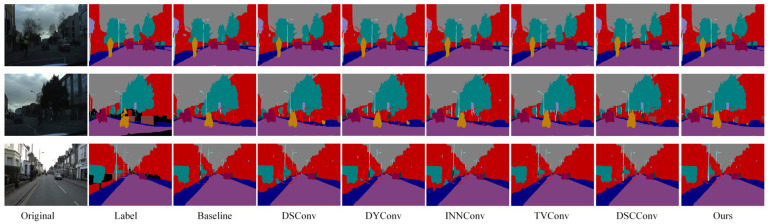
Qualitative comparison experimental results of different convolution modules on the CamVid dataset.

As shown in the red-circled region of Figure 9, the model employing spider web convolution demonstrates more accurate and complete recognition of building areas compared to the baseline and other convolution modules. It exhibits significantly fewer misclassifications, clearer edge contours, and finer detail preservation, further illustrating its superior feature representation capability in complex scenes.

To evaluate the performance of different convolutional structures in complex scenarios, Table 7 presents a quantitative comparison conducted on the BDD100K dataset. It is evident that the proposed SWConv module outperforms other convolutional variants in more challenging autonomous driving environments. Specifically, compared to the DSConv module, SWConv achieves improvements of 1.26%, 0.70%, 1.63%, and 0.27% in mIoU, mRecall, mPrecision, and mAccuracy, respectively. These results indicate that SWConv is more effective in extracting key regional features, enhancing the accuracy and completeness of target recognition, and thereby improving the model’s practical adaptability to complex real-world conditions.

**Figure 10:**
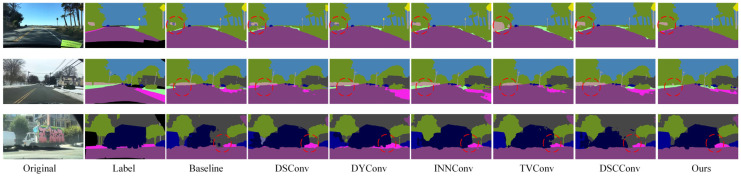
Qualitative comparison experimental results of different convolution modules on the BDD100K dataset.

In addition to the quantitative comparison, we also conducted qualitative visualization analysis to more comprehensively evaluate the performance of different convolution structures in complex scenarios. As shown in Figure 10, we selected several representative challenging environments to visually compare the segmentation results of each module. The results indicate that the model based on the SWConv module produces more complete and coherent segmentation maps in key regions, exhibiting superior structural consistency and semantic accuracy. This further highlights the advantages of SWConv in feature extraction, as well as its strong robustness in complex real-world environments, demonstrating its effectiveness in adapting to diverse scene variations.

To further validate the advantages of the proposed module in convolutional structure selection, this paper conducts a comparative analysis of segmentation models replacing different types of convolutional operators in terms of parameter count and FLOPs. The experimental results are shown in Table 8. It can be observed that, compared to most convolutions, the proposed SWConv module incurs some increase in computational cost due to its padding branch design, but remains within a reasonable and acceptable range. Among these, configurations with only two padding branches and four padding branches (e.g., SW-UD padding the up and down directions, SW-UDLF padding up, down, left, and right directions) demonstrate clear computational advantages over TVConv and DSCConv. Meanwhile, SWConv significantly improves segmentation performance while maintaining a reasonable computational burden, exhibiting superior representational capacity and feature extraction capabilities compared to other convolution modules. Furthermore, considering the strict constraints on computational resources and power consumption in embedded devices, the advantages of the SWConv module in parameters and FLOPs provide a solid foundation for its practical deployment. Its balanced computational cost and efficient feature representation endow SWConv with strong adaptability, enabling it to meet the dual requirements of real-time performance and energy efficiency in embedded systems, thereby demonstrating promising application potential.

#### 4.2.3. Comparison of Experimental Performance of Other Methods

The above experimental analysis shows that integrating the global–local collaborative attention module and the spider convolution module into the baseline model significantly improves semantic segmentation performance across multiple evaluation metrics. To further evaluate the overall performance of the final model in the context of autonomous driving, we conducted comparative experiments with several classic semantic segmentation models. Since different methods apply image cropping with varying sizes during training on the Cityscapes and BDD100K datasets, a comparative evaluation was conducted on the CamVid dataset (resolution 480 × 360) to ensure fairness and consistency. The models compared include SegNet [43], Enet [44], ESCNet [19], Algorithm [14], NDNet [45], LEANet [46], EDANet [47], CGNet [17], DABNet [48], LRDNet [49], DSANet [18], MSCFNet [50], and the benchmark model DeepLabv3+. The results for each model are obtained from their original publication. The experimental results are presented in Table 9, where N denotes values that are either unknown or not reported. All experimental equipment was provided by NVIDIA Corporation, headquartered in Santa Clara, California, USA.

To better highlight the performance differences between methods, this study provides a detailed analysis of all compared models and discloses the devices, training configurations, and parameter settings used, ensuring transparency and comparability of results. Notably, while the original baseline model performed worse than LRDNet, DSANet, and MSCFNet, integrating the GLCA and SWConv modules led to a significant performance improvement, surpassing all other models.

As shown in Table 9, the final model enhanced with the two proposed modules achieves the best performance across all evaluation metrics, demonstrating its strong capability in semantic segmentation for autonomous driving. The model significantly outperforms other approaches, with the mIoU improving by 0.35% compared to the second-best model. This improvement is not only a testament to the effectiveness of the GLCA module in enhancing feature interaction across different spatial scales but also to the spider web convolution’s ability to capture complex structural information in diverse environments. By effectively integrating global and local feature representations, the model exhibits a much better capacity to handle the intricate and variable nature of real-world driving scenes. These results underscore the strong potential of the proposed modules to enhance segmentation accuracy while maintaining efficient computation, making the model suitable for practical applications in autonomous driving systems.

### 4.3. Ablation Experiment

#### 4.3.1. Ablation Study on GLCA and SWConv Modules

In the ASPP module, convolutions with different dilation rates and standard convolutions are used to capture multi-scale contextual information. The first layer of the ASPP module employs a standard 1 × 1 convolution, which has the smallest receptive field and mainly serves to retain fine-grained local texture information. However, it is limited in terms of semantic modeling. The second and third layers use dilated convolutions with rates of 6 and 12, respectively, providing medium-to-relatively large receptive fields. These layers strike a balance between capturing local details and fusing contextual semantics. The fourth layer employs a dilated convolution with a rate of 18, providing the largest receptive field and is thus better suited for capturing wide-range, global semantic information. However, as the receptive field increases, the sampling points of the convolution become increasingly sparse. This sparsity, especially in the rate = 18 path, impairs the model’s ability to perceive local details and object boundaries, making it difficult to model targets with complex shapes or irregular structures.

To address this issue, we incorporate the GLCA and SWConv modules into the rate = 18 path of the ASPP module, aiming to enhance this path’s capacity for semantic fusion and structural modeling. The GLCA module leverages bidirectional feature interaction and a dynamic weighting mechanism to effectively integrate global semantics with local detail, enhancing the representation of key regions and mitigating issues such as semantic ambiguity and blurred boundaries. This is particularly beneficial for the rate = 18 path, where the receptive field is large but information is sparse. The SWConv module introduces an asymmetric sampling topology and six-directional perception paths, breaking conventional convolution’s reliance on regular structures. This improves the model’s ability to capture object shapes, edge contours, and spatial structures, thereby compensating for the rate = 18 path’s deficiencies in shape adaptability. In contrast, the 1 × 1 convolution path and low-to-medium rate dilated convolution paths already possess strong capabilities in capturing local features due to their denser structures. As a result, the benefits of adding GLCA and SWConv to these paths are relatively limited. The rate = 18 path, however, combines high-level semantic abstraction with a significant information gap, making it the optimal location for the combined application of both modules.

To evaluate the individual effectiveness and synergistic adaptability of the proposed GLCA and SWConv modules in semantic segmentation, we conducted a systematic ablation study. Table 10 presents the results on the Cityscapes dataset. The two modules were inserted into or used to replace different components of the ASPP module under two input resolutions (256 × 256 and 768 × 768). Specifically, the positions labeled 1, 6, 12, and 18 correspond to the first feature extraction layer and the dilated convolution layers with dilation rates of 6, 12, and 18 in the ASPP module, respectively.

Experimental results show that regardless of the insertion position within the ASPP module, both the GLCA and SWConv modules consistently improve performance. Notably, placing the GLCA module and SWConv module in the fourth layer of the ASPP module (rate = 18) yields the best results across all evaluation metrics. This indicates that introducing these modules at the final stage of feature extraction helps integrate the multi-scale semantic information captured by the preceding layers more effectively, thereby enhancing the model’s ability to capture cross-layer feature dependencies.

Furthermore, to further validate the synergistic benefits of the two modules, we integrated both the GLCA module and the SWConv module into the final layer of the ASPP module. Comparative experimental results demonstrate that the combined use of these modules leads to significantly better performance than using either module alone. At an input resolution of 256 × 256, the model achieved an improvement of 2.73% in mIoU, 2.64% in mRecall, 2.15% in mPrecision, and 0.27% in mAccuracy. At a higher resolution of 768 × 768, mIoU increased by 1.47%, mRecall by 1.80%, mPrecision by 0.32%, and mAccuracy by 0.13%. Based on the above quantitative experimental results, the proposed GLCA and SWConv modules demonstrate superior performance across different input resolutions, validating their strong scale adaptability and cross-scene generalization capability. These modules consistently and stably enhance model performance in multi-resolution semantic segmentation tasks.

**Figure 11:**
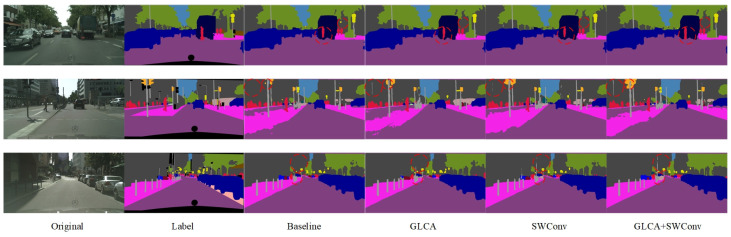
Qualitative ablation comparison experimental results of GLCA and SWConv modules on the Cityscapes dataset.

Figure 11 presents the semantic segmentation visualization results on the Cityscapes dataset, showing the step-by-step integration of the GLCA and SWConv modules into the baseline model. As highlighted in the annotated regions of the figure, the inclusion of these two modules leads to more accurate recognition of fine-grained objects such as pedestrians, poles, and traffic lights. The object boundaries become noticeably clearer, misclassifications are significantly reduced, and the segmentation results show substantial improvements in both completeness and precision. These visual results further validate the synergistic effect of the GLCA and SWConv modules, demonstrating that they not only enhance the model’s capability in object perception and boundary localization but also effectively mitigate semantic confusion across categories.

To validate the stability and generalization of the proposed modules across different scenarios and resolutions, additional experiments were conducted on the CamVid dataset. Since no complex cropping and restoration operations were performed during training and testing at 480 × 360 resolution, the FPS metric is introduced to better assess the real-time processing capability of the model and its modules. Table 11 shows the ablation experiment results of the GLCA and SWConv modules on the CamVid dataset, tested at two resolutions, 480 × 360 and 960 × 720. The experiments show that the two proposed modules significantly improve model performance when integrated into the ASPP module at various positions, with the best effect achieved when placed at the dilation convolution with a dilation rate of 18. At a resolution of 480 × 360, after introducing the two modules, the mIoU improves by 1.29%, mRecall by 0.64%, and mPrecision and mAccuracy by 1.81% and 0.34%, respectively, compared to the baseline model. At a high resolution of 960 × 720, mIoU is improved by 1.48%, mRecall by 1.95%, and mAccuracy by 0.26%, while mPrecision is optimal after replacing it with the SWConv module. The overall experimental results further verify the effectiveness of the GLCA and SWConv modules in enhancing model performance, particularly demonstrating stronger stability and adaptability under high-resolution input. Both modules bring performance gains at different insertion positions, with the combined use yielding the most significant effect, reflecting good module compatibility and complementarity. It is worth mentioning that although there is a slight sacrifice in inference speed, the accuracy is significantly improved, demonstrating a better trade-off between accuracy and efficiency.

**Figure 12:**
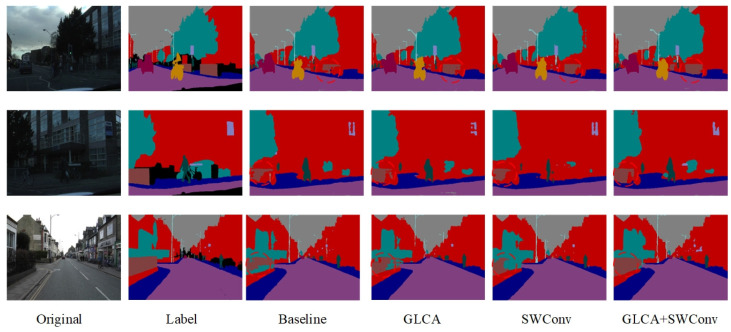
Qualitative ablation comparison experimental results of GLCA and SWConv modules on the CamVid dataset.

Figure 12 shows the semantic segmentation results on the CamVid dataset after gradually adding modules. As seen in the marked area, the baseline model exhibits noticeable semantic confusion and boundary discontinuities, especially around structures like walls, indicating its limited ability to capture fine-grained details. With the introduction of the GLCA and SWConv modules, the model’s ability to understand semantics and accurately locate boundaries improves significantly, with the segmentation results progressively refining from coarse to fine. Finally, when both modules are integrated, they not only reduce semantic interference between categories but also enhance the clarity and integrity of structural edges. The segmented images become visually closer to the true labels, both in terms of appearance and structural consistency.

Table 12 presents the quantitative ablation results on the BDD100K dataset. It can be observed that, based on the baseline segmentation model, progressively introducing the GLCA and SWConv modules leads to improvements across all four evaluation metrics, fully demonstrating the effectiveness and applicability of these two modules in complex autonomous driving scenarios. When the GLCA and SWConv modules are integrated simultaneously, the segmentation performance is further enhanced. Specifically, compared to the baseline model, mIoU increases by 1.73%, mRecall by 1.82%, mPrecision by 1.62%, and mAccuracy by 0.32%. These results indicate that the proposed modules work synergistically to effectively enhance the model’s ability to perceive and recognize diverse semantic information in autonomous driving environments, thereby improving overall segmentation performance and robustness.

**Figure 13:**
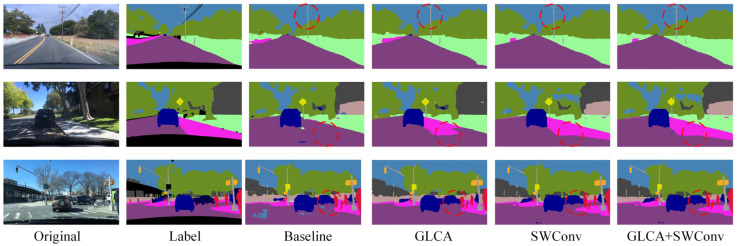
Qualitative ablation comparison experimental results of GLCA and SWConv modules on the BDD100K dataset.

To more intuitively verify the performance improvements brought by the proposed modules in complex scenarios, qualitative ablation experiments were conducted on the BDD100K dataset. Figure 13 shows a comparison of segmentation results from the baseline model, the model with the GLCA module, the model with the SWConv module, and the model combining both modules. The baseline model exhibits semantic ambiguity and unclear boundaries in some detailed regions. After introducing the GLCA module, the model achieves more accurate feature representation in key local areas, enhancing its ability to capture small objects and fine details. The addition of the SWConv module strengthens the model’s perception of multi-directional spatial information, effectively improving the representation of object contours and shapes. When both modules are combined, the model maintains overall semantic consistency while producing finer details and more precise boundaries, significantly enhancing the visual quality of the segmentation results. This qualitative analysis further validates the complementary advantages and practical effectiveness of the GLCA and SWConv modules in complex autonomous driving scenarios.

**Figure 14:**
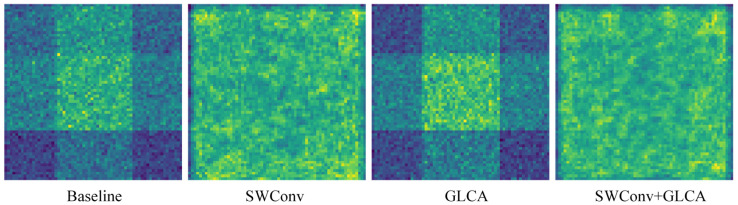
Feature map changes and representations after the introduction of SWConv and GLCA modules.

To better understand the function of each module, we will next demonstrate the specific changes in the feature maps after introducing different modules and analyze their impact on the model’s performance. The feature map results are shown in Figure 14. It can be observed that after introducing SWConv, the feature extraction performance is significantly improved compared to the original feature map. In the original feature map, the effective features in the bright regions are scattered, and multi-directional information is not fully integrated, leading to blurred target boundaries and spatial relationships. After adopting SWConv, the module integrates multi-directional contextual information, making the bright regions in the feature map more coherent and providing more comprehensive coverage. Features such as road markings, vehicle contours, and pedestrian trajectories are more precisely presented, enhancing the perception of target boundaries and spatial relationships, providing more effective feature support for tasks in autonomous driving scenarios. After introducing the GLCA module, the key regions in the feature map become more prominent, indicating a significant increase in feature concentration in those areas. The GLCA module enhances the model’s ability to focus on critical regions of the image by integrating global and local contextual information. It dynamically adjusts the importance of these areas, thereby increasing attention on key features. This improvement enables the model to more accurately recognize fine details and comprehend spatial relationships, thereby enhancing its ability to capture the essential aspects of the scene.

Finally, after using both modules together, the performance is further improved. The attention module complements SWConv in global context understanding and feature correlation capture, allowing SWConv to focus more on the key features and strengthening the connection between various parts of the feature map. The combination of the attention module and SWConv enhances feature extraction, making it more comprehensive and precise, as they work synergistically to optimize the model’s recognition and decision-making capabilities in autonomous driving.

#### 4.3.2. Ablation Study on Hyperparameters of GLCA and SWConv

To comprehensively evaluate the impact of key module designs on the overall performance of the segmentation model, we further analyze the role of core hyperparameters. This work conducts an extensive experimental analysis on the design of the local feature extraction branch, global feature extraction branch, and dynamic weighting branch in the GLCA module, as well as the hyperparameter of padding direction numbers in the SWConv module. By systematically adjusting these parameters and observing the resulting performance changes, we can clarify the performance boundaries and stability of each structural design. Here, GLCA-NL, GLCA-NG, and GLCA-NW represent the removal of the local feature extraction branch, global feature extraction branch, and the absence of the dynamic weighting branch, wherein the latter adopts equal-weight averaging for feature fusion. SWConv-UD, SWConv-LF, SWConv-TD, SWConv-UDTD, SWConv-LFTD, and SWConv-UDLF denote configurations using only the up-down padding branch, left-right padding branch, diagonal padding branch, up-down plus diagonal branches, left-right plus diagonal branches, and up-down, left-right plus diagonal branches, respectively. To systematically assess and analyze these components, experiments are conducted on the Cityscapes and CamVid datasets.

The quantitative experimental results of each hyperparameter component on the Cityscapes dataset are shown in Table 13. From the data observed in the table, it is evident that removing the local feature extraction branch, global feature extraction branch, or dynamic weighting branch in the GLCA module leads to a certain degree of performance degradation across metrics such as mIoU, mRecall, mPrecision, and mAccuracy, indicating that all branches play a crucial role in feature extraction and fusion. Notably, removing the local feature extraction branch results in the most severe decline, with decreases of 1.75%, 1.65%, 1.79%, and 0.08% in mIoU, mRecall, mPrecision, and mAccuracy, respectively. The quantitative experiments demonstrate that the hyperparameter configurations of these branch structures have a significant impact on model performance, further underscoring the importance of jointly optimizing structural design and hyperparameter settings in practical applications. Similarly, in the SWConv module, removing different numbers of padding branches also leads to significant performance drops, illustrating that the choice of padding branch quantity has a critical impact on feature representation capability. Various combinations of padding branch numbers and directions capture diverse spatial information and directional features, enhancing the model’s sensitivity to complex scene details. However, the impact of different padding direction combinations varies, as unreasonable combinations may cause information redundancy or reduce feature representation efficiency, resulting in performance degradation. Compared to SWConv-UD, the SWConv module improves mIoU, mRecall, mPrecision, and mAccuracy by 0.97%, 0.65%, 1.10%, and 0.17%, respectively.

**Figure 15:**
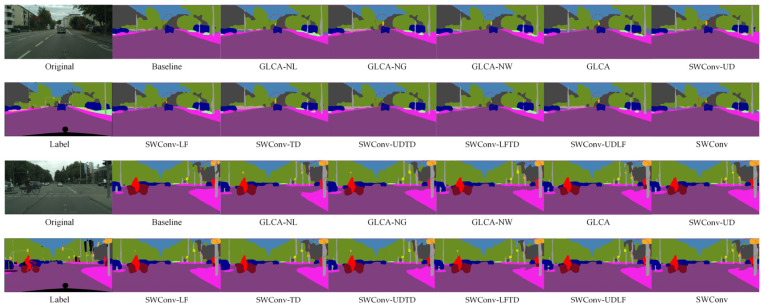
Qualitative results of ablation experiments on the hyperparameters of GLCA and SWConv modules on the Cityscapes dataset.

The quantitative experimental results of each hyperparameter component on the Cityscapes dataset are illustrated in Figure 15. From the visualization results, it can be further observed that different hyperparameter components have a significant impact on model performance. Through reasonable hyperparameter tuning, the model achieves more comprehensive learning of edge details and small object regions, yielding superior segmentation results. Compared to other hyperparameter module designs, the final GLCA and SWConv modules proposed in this work demonstrate exceptional performance in capturing fine-grained features and restoring complex scene details, effectively enhancing overall segmentation accuracy and robustness.

**Figure 16:**
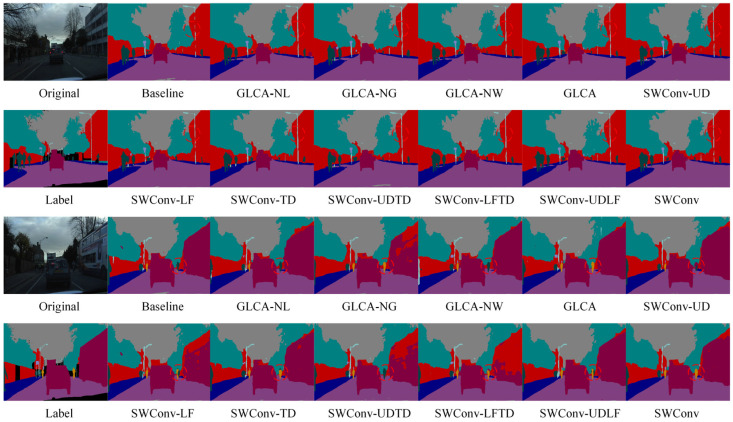
Qualitative results of ablation experiments on the hyperparameters of GLCA and SWConv modules on the CamVid dataset.

To evaluate the robustness and adaptability of different hyperparameters across various scenarios, we also conducted experiments on the CamVid dataset, with the quantitative experimental results presented in Table 14. The results indicate that the hyperparameter configurations of different components maintain consistent performance trends on this dataset, further validating the effectiveness and generalizability of the proposed modular design. Specifically, in the GLCA module, the full GLCA configuration outperforms GLCA-NL by 1.43%, 1.08%, 2.20%, and 0.36% in terms of mIoU, mRecall, mPrecision, and mAccuracy, respectively. In the SWConv module, our SWConv configuration achieves improvements of 0.96%, 0.55%, 1.22%, and 0.33% over SWConv-LFTD across the same metrics. The qualitative results are shown in Figure 16. From the visual comparisons, it can be observed that both the GLCA and SWConv modules produce finer segmentation results, particularly in edge contours, small object regions, and structurally complex scenes. They demonstrate a superior ability to preserve semantic boundaries and exhibit better detail recovery and visual consistency compared to other hyperparameter settings, further confirming their effectiveness and adaptability across diverse scenarios.

## 5. Discussion

### 5.1. Model Innovations and Advantages

This paper proposes novel GLCA and SWConv modules for autonomous driving perception tasks, aiming to enhance the model’s ability to represent diverse semantic targets and improve segmentation accuracy in complex road scenes. Our work also effectively addresses common challenges in semantic segmentation, such as blurred boundary delineation and difficulty in capturing irregular objects, thereby providing new module design ideas and methodological approaches for computer vision research focused on object detection and scene understanding.

We conducted systematic evaluations on three authoritative public datasets, Cityscapes, CamVid, and BDD100k. Both datasets were captured by vehicle-mounted sensors, realistically reflecting the visual complexity faced by camera perception systems in urban driving scenarios and representing typical real-world application environments. Experimental results demonstrate that the model achieves significant improvements across multiple key metrics, including mIoU, mRecall, mPrecision, and mAccuracy. Further visualization analysis shows that, when confronted with complex traffic scenarios and dynamically changing road conditions, the improved model can more accurately distinguish key categories such as lane lines, traffic signs, vehicles, and pedestrians. The optimized model enhances the understanding of complex dynamic scenes and real-time processing capabilities, which is of great significance for improving the safety and reliability of ADAS systems in real-world traffic environments.

### 5.2. Limitations and Future Work

This study improves semantic segmentation accuracy while maintaining a certain level of computational efficiency by introducing the lightweight backbone MobileNetV2, along with the independently designed GLCA and SWConv modules. However, there remain aspects that require further optimization. First, the performance gains come with additional computational overhead, resulting in reduced inference speed compared to the baseline model, reflecting the inherent trade-off between accuracy and efficiency. Future research may explore various model compression strategies, such as applying post-training quantization or quantization-aware training to reduce storage and computational demands; employing structured pruning to remove redundant convolutional channels and enhance runtime efficiency; or leveraging structural re-parameterization techniques to simplify the network architecture during inference while preserving model expressiveness, thereby improving deployability in real-time applications.

Moreover, the model’s robustness in complex or dynamically changing scenarios still has room for improvement. Subsequent work could consider constructing more expressive multi-level semantic fusion architectures and incorporating knowledge distillation techniques to transfer the representational capacity of large-scale models into the current lightweight framework, further optimizing the balance between performance and efficiency. Meanwhile, the current study has not systematically evaluated the model’s performance under real-world environmental conditions such as nighttime driving, adverse weather, and varying lighting. Nighttime driving often suffers from low illumination, resulting in reduced signal-to-noise ratio and loss of edge and texture details, which increases the difficulty of semantic segmentation. Adverse weather conditions, such as rain, fog, and snow, introduce image blurring, occlusion, and decreased contrast, affecting the model’s ability to accurately recognize objects. Different lighting conditions (e.g., backlighting, strong shadows, or artificial illumination) may cause color shifts and localized overexposure or underexposure, interfering with the model’s feature extraction capabilities. Future work will focus on designing specialized datasets and evaluation metrics for these complex environments, integrating multimodal information fusion, enhancement learning, and adaptive mechanisms to improve model adaptability and reliability under varying conditions, thereby meeting the demands of practical applications.

Finally, the datasets used in this study remain limited in scale and diversity, which may affect the model’s generalization capability. Future efforts will further explore richer data augmentation strategies and image generation techniques to increase the diversity of training samples, thereby enhancing the model’s adaptability and stability in variable environments.

## 6. Conclusions

This study focuses on the semantic segmentation task in autonomous driving. It proposes the global–local collaborative attention module and spider web convolution module, designing a segmentation network architecture suitable for complex environments. By achieving pixel-level classification of road scenes, our model provides technical support for key downstream tasks such as path planning, obstacle avoidance, and traffic signal recognition, thereby contributing to the enhancement of safety, reliability, and intelligence in autonomous vehicles. Experimental results demonstrate significant improvements across multiple key metrics, achieving a good balance among accuracy, efficiency, and module versatility. Validation on different datasets and resolutions further confirms the robustness and adaptability of the method, providing solid support for the practical deployment of autonomous driving perception systems. Future research can build upon this work to further advance the intelligent development of autonomous driving perception systems.

## Figures and Tables

**Table 1:** Quantitative comparative experimental results of different attention mechanisms and transformer modules on the Cityscapes dataset.

Input Size	Method	mIoU	mRecall	mPrecision	mAccuracy
256 × 256	Baseline	54.57	66.23	71.06	90.92
	SE [27]	55.68	67.20	71.77	91.10
	CBAM [28]	55.75	67.78	71.44	91.08
	SK [29]	56.09	67.69	72.14	91.04
	CA [30]	56.32	68.20	72.17	91.05
	ECA [31]	56.01	67.58	72.20	91.02
	BAM [32]	56.23	67.78	72.15	90.99
	Swin-TF [39]	56.45	68.30	72.17	91.09
	HAT-Net [40]	56.30	67.91	72.56	91.06
	GLCA	**56.79**	**68.34**	**72.64**	**91.15**

**Table 2:** Quantitative comparative experimental results of different attention mechanisms and transformer modules on the CamVid dataset.

Input Size	Method	mIoU	mRecall	mPrecision	mAccuracy
480 × 360	Baseline	68.99	76.96	84.05	92.19
	SE [27]	69.11	76.47	**85.27**	92.27
	CBAM [28]	68.74	76.12	84.45	92.20
	SK [29]	69.18	76.50	84.84	92.32
	CA [30]	68.99	76.65	83.84	92.20
	ECA [31]	69.75	77.46	84.35	92.20
	BAM [32]	68.96	76.33	84.53	92.30
	Swin-TF [39]	69.38	76.85	84.32	92.49
	HAT-Net [40]	68.88	76.46	84.24	92.31
	GLCA	**69.96**	**77.54**	85.04	**92.50**

**Table 3:** Quantitative comparative experimental results of different attention mechanisms and transformer modules on the BDD100K dataset.

Input Size	Method	mIoU	mRecall	mPrecision	mAccuracy
512 × 512	Baseline	56.06	67.65	69.53	92.09
	SE [27]	56.65	68.25	70.20	92.18
	CBAM [28]	56.14	67.01	70.42	92.11
	SK [29]	56.26	67.93	70.28	92.14
	CA [30]	56.24	68.73	69.19	92.18
	ECA [31]	56.33	68.73	69.76	92.03
	BAM [32]	56.35	68.78	69.25	91.71
	Swin-TF [39]	56.68	68.12	70.31	92.16
	HAT-Net [40]	56.48	69.09	69.38	92.09
	GLCA	**57.09**	**69.14**	**70.45**	**92.18**

**Table 4:** Comparative analysis of parameters (Params/M) and FLOPs (FLOPs/G) in models integrating various attention mechanisms and transformer modules.

Method	SE	CBAM	SK	CA	ECA	BAM	Swin-TF	HAT-Net	GLCA
Params	5.826	5.831	11.37	5.825	5.818	5.839	6.608	6.615	5.971
FLOPs	26.40	26.41	41.27	26.40	26.40	26.43	28.52	28.54	26.60

**Table 5:** Quantitative comparison experimental results of different convolution modules on the Cityscapes dataset.

Input Size	Method	mIoU	mRecall	mPrecision	mAccuracy
256 × 256	Baseline	54.57	66.23	71.06	90.92
	DSConv [33]	56.21	68.64	71.22	91.06
	DYConv [34]	56.36	68.58	71.76	91.06
	INNConv [35]	55.86	67.85	71.37	91.10
	TVConv [41]	55.17	66.46	71.52	90.98
	DSCConv [42]	55.83	67.61	71.63	90.98
	SWConv	**56.92**	**68.85**	**72.51**	**91.19**

**Table 6:** Quantitative comparison experimental results of different convolution modules on the CamVid dataset.

Input Size	Method	mIoU	mRecall	mPrecision	mAccuracy
480 × 360	Baseline	68.99	76.96	84.05	92.19
	DSConv [33]	69.28	76.77	84.51	92.24
	DYConv [34]	68.95	77.05	83.45	92.08
	INNConv [35]	68.37	76.16	83.53	92.11
	TVConv [41]	69.41	76.91	84.37	92.39
	DSCConv [42]	69.47	77.30	83.86	92.23
	SWConv	**70.01**	**77.31**	**85.25**	**92.45**

**Table 7:** Quantitative comparison experimental results of different convolution modules on the BDD100K dataset.

Input Size	Method	mIoU	mRecall	mPrecision	mAccuracy
512 × 512	Baseline	56.06	67.65	69.53	92.09
	DSConv [33]	56.14	67.92	69.47	92.13
	DYConv [34]	56.84	67.84	70.81	92.30
	INNConv [35]	56.73	68.25	70.94	92.08
	TVConv [41]	57.14	68.23	71.07	92.38
	DSCConv [42]	56.93	68.50	70.82	92.36
	SWConv	**57.40**	**68.62**	**71.10**	**92.40**

**Table 8:** Comparative analysis of parameters (Params/M) and FLOPs (FLOPs/G) in models incorporating different convolutional structures.

Convs	DS	DY	INN	TV	DSC	SW-UD	SW-UDLR	SW
Params	5.166	5.107	5.189	8.317	6.396	5.459	5.836	7.443
FLOPs	24.63	24.41	24.70	29.41	27.96	26.15	27.88	32.41

**Table 9:** Comparative experimental results of various semantic segmentation models on the CamVid dataset.

Input Size	Method	Device	Pre-trained	mIoU
480 × 360	SegNet [43]	Titan X	ImageNet	60.10
	Enet [44]	Titan X	N	51.30
	ESCNet [19]	Titan X	N	56.10
	Algorithm [14]	GTX 1070	N	55.95
	NDNet [45]	Titan X	N	57.20
	LEANet [46]	RTX 1080Ti	N	67.50
	EDANet [47]	N	N	66.40
	CGNet [17]	V100	N	65.60
	DABNet [48]	RTX 1080Ti	N	66.40
	LRDNet [49]	GTX 1080Ti	N	69.70
	DSANet [18]	GTX1080	N	69.93
	MSCFNet [50]	N	N	69.30
	Deeplabv3+ [12]	RTX 4090	ImageNet	68.99
	Ours	RTX 4090	ImageNet	**70.28**

**Table 10:** Quantitative results of ablation experiments on each module at various embedding positions in the Cityscapes dataset at different resolutions.

Input Size	Method	mIoU	mRecall	mPrecision	mAccuracy
256 × 256	Baseline	54.57	66.23	71.06	90.92
	GLCA-1	56.39	68.23	72.35	90.99
	GLCA-6	56.31	68.42	71.86	91.00
	GLCA-12	56.56	68.65	72.10	91.12
	GLCA-18	56.79	68.34	72.64	91.15
	SWConv-1	56.62	68.26	72.50	91.19
	SWConv-6	56.32	68.31	72.01	90.96
	SWConv-12	56.14	68.05	71.73	91.05
	SWConv-18	56.92	68.85	72.51	91.19
	Ours	**57.30**	**68.87**	**73.21**	**91.19**
768 × 768	Baseline	71.79	81.35	84.37	94.76
	GLCA-18	73.10	82.61	**84.91**	94.86
	SWConv-18	72.31	82.24	84.14	94.73
	Ours	**73.26**	**83.15**	84.69	**94.89**

**Table 11:** Quantitative results of ablation experiments on each module at various embedding positions in the CamVid dataset at different resolutions.

Input Size	Method	mIoU	mRecall	mPrecision	mAccuracy	FPS
480 × 360	Baseline	68.99	76.96	84.05	92.19	**65.95**
	GLCA-1	69.60	76.92	85.81	92.42	60.55
	GLCA-6	69.03	76.95	83.17	92.08	60.57
	GLCA-12	69.06	76.19	85.19	92.37	60.54
	GLCA-18	69.96	77.54	85.04	92.50	60.41
	SWConv-1	69.31	76.65	84.76	92.44	62.54
	SWConv-6	69.56	77.41	84.16	92.33	61.43
	SWConv-12	69.29	76.58	85.06	92.46	61.41
	SWConv-18	70.01	77.31	85.25	92.45	61.52
	Ours	**70.28**	**77.60**	**85.86**	**92.53**	55.79
960 × 720	Baseline	72.77	79.07	88.54	93.36	**32.98**
	GLCA-18	74.07	80.89	88.02	93.54	32.34
	SWConv-18	73.10	79.45	**88.75**	93.43	31.54
	Ours	**74.25**	**81.02**	88.07	**93.62**	29.63

**Table 12:** Quantitative ablation comparison experimental results of GLCA and SWConv modules on the BDD100K dataset.

Input Size	Method	mIoU	mRecall	mPrecision	mAccuracy
512 × 512	Baseline	56.06	67.65	69.53	92.09
	GLCA-18	57.09	69.14	70.45	92.18
	SWConv-18	57.40	68.62	71.10	92.40
	Ours	**57.79**	**69.47**	**71.15**	**92.41**

**Table 13:** Quantitative results of ablation experiments on the hyperparameters of GLCA and SWConv modules on the Cityscapes dataset.

Input Size	Method	mIoU	mRecall	mPrecision	mAccuracy
256 × 256	Baseline	54.57	66.23	71.06	90.92
	GLCA-NL	55.04	66.69	70.85	91.07
	GLCA-NG	56.23	67.80	72.31	91.03
	GLCA-NW	56.25	67.89	72.08	91.14
	GLCA	**56.79**	**68.34**	**72.64**	**91.15**
	SWConv-UD	55.95	68.20	71.41	91.02
	SWConv-LF	56.12	67.76	71.98	91.08
	SWConv-TD	56.48	68.50	72.12	91.05
	SWConv-UDTD	55.72	67.50	71.69	91.03
	SWConv-LFTD	56.15	67.85	72.17	91.14
	SWConv-UDLF	56.24	67.81	72.32	91.04
	SWConv	**56.92**	**68.85**	**72.51**	**91.19**

**Table 14:** Quantitative results of ablation experiments on the hyperparameters of GLCA and SWConv modules on the CamVid dataset.

Input Size	Method	mIoU	mRecall	mPrecision	mAccuracy
480 × 360	Baseline	68.99	76.96	84.05	92.19
	GLCA-NL	68.53	76.46	82.84	92.14
	GLCA-NG	69.11	76.81	84.29	92.32
	GLCA-NW	69.35	77.06	83.74	92.20
	GLCA	**69.96**	**77.54**	**85.04**	**92.50**
	SWConv-UD	69.67	77.28	84.73	92.37
	SWConv-LF	69.25	76.73	84.09	92.30
	SWConv-TD	69.42	76.78	84.73	92.45
	SWConv-UDTD	69.84	77.30	85.08	92.37
	SWConv-LFTD	69.05	76.76	84.03	92.12
	SWConv-UDLF	69.37	76.64	84.62	92.45
	SWConv	**70.01**	**77.31**	**85.25**	**92.45**

## Data Availability

Cityscapes, CamVid, and BDD100K are publicly available datasets and were analyzed in this study. These datasets can be accessed here: https://www.cityscapes-dataset.com (accessed on 27 March 2025), https://github.com/alexgkendall/SegNet-Tutorial/tree/master/CamVid (accessed on 2 April 2025) and http://bdd-data.berkeley.edu/ (accessed on 17 July 2025).
